# Supplementary material for: Efficacy and safety of electric acupuncture in treatment of intractable facial paralysis: A protocol for systematic review and meta-analysis
Source: PLoS One. 2022 Dec 1;17(12):e0278509. doi: 10.1371/journal.pone.0278509 (PMC9714806; doi:10.1371/journal.pone.0278509)
Supplement: S2 Appendix — (DOCX) [file pone.0278509.s002.docx]

Table 1. The search strategy for PubMed.

| **Order** | **strategy** |
| --- | --- |
| #1 | Search: “Facial paralysis”[Mesh] |
| #2 | Search: “Paralyses, Facial”[Title/Abstract] OR “Facial Palsy”  [Title/Abstract] OR “Intractable facial paralysis”[Title/Abstract] OR “Facial nerve paralysis”[Title/Abstract] OR “Facial nerve disease”  [Title/Abstract] |
| #3 | #1 OR #2 |
| #4 | Search: “Electroacupuncture”[Title/Abstract] OR “Electrical acupuncture”[Title/Abstract] OR “Electric acupuncture”[Title/Abstract] OR “Galvanoacupuncture”[Title/Abstract] OR “EA, Electroacupuncture”  [Title/Abstract] |
| #5 | Search: “randomized controlled trial”[Publication Type] OR “RCT randomized controlled”[Publication Type] OR “random allocation”[Title/Abstract] OR “allocation, random”[Title/Abstract] OR “randomized, controlled”[Title/Abstract] OR “clinical trial”[Title/Abstract] |
| #6 | Search: “humans”[MeSH Terms] NOT “animals”[MeSH Terms] |
| #7 | #5 AND #6 |
| #8 | #3 AND #4 AND #7 |
