# Supplementary figures and images for: Efficacy and safety of electric acupuncture in treatment of intractable facial paralysis: A protocol for systematic review and meta-analysis
Source: PLoS One. 2022 Dec 1;17(12):e0278509. doi: 10.1371/journal.pone.0278509 (PMC9714806; doi:10.1371/journal.pone.0278509)

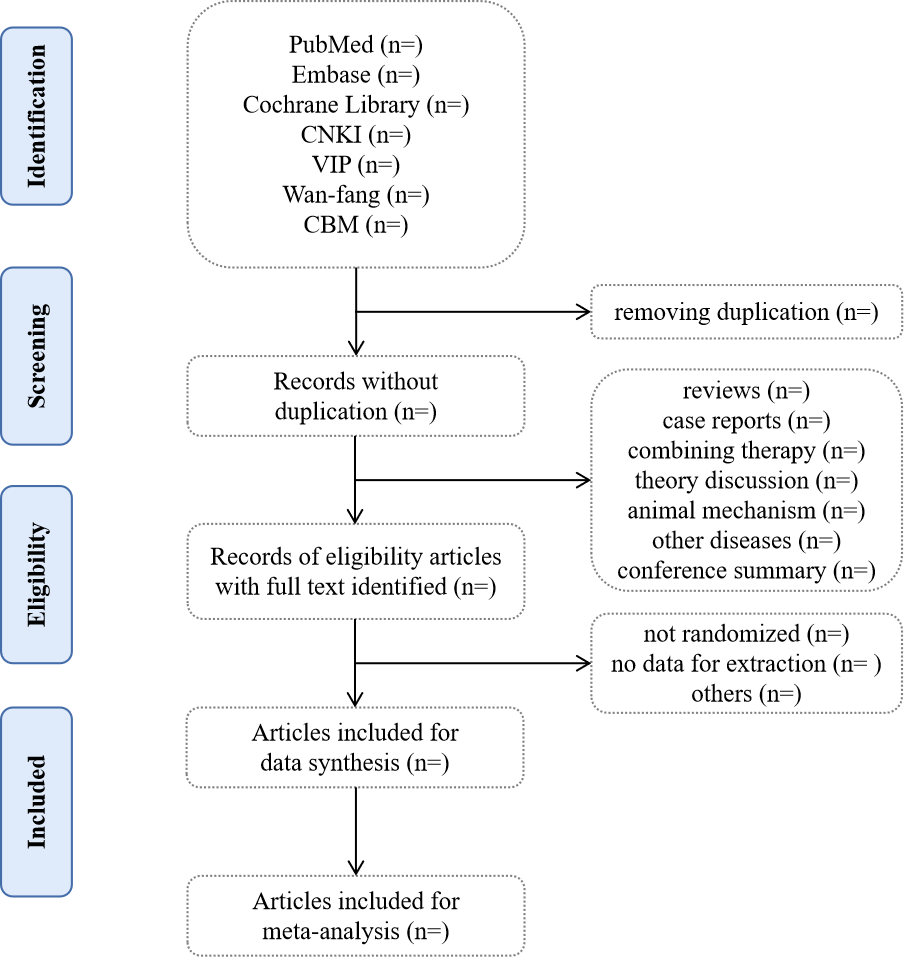

Supplement: S1 Fig — (TIF) [file pone.0278509.s003.tif]
